# Tracing the source of infection of cystic and alveolar echinococcosis, neglected parasitic infections with long latency: The shaky road of “evidence” gathering

**DOI:** 10.1371/journal.pntd.0009009

**Published:** 2021-01-21

**Authors:** Adriano Casulli, Francesca Tamarozzi

**Affiliations:** 1 WHO Collaborating Centre on Epidemiology, Detection and Control of Cystic and Alveolar Echinococcosis (in Animals and Humans), European Union Reference Laboratory for Parasites, Department of Infectious Diseases, Istituto Superiore di Sanità, Rome, Italy; 2 Department of Infectious Tropical Diseases and Microbiology, IRCCS Sacro Cuore Don Calabria Hospital, Negrar di Valpolicella, Verona, Italy; PUCRS, BRAZIL

We read with interest the work by Torgerson and colleagues “Source attribution of human echinococcosis: a systematic review and meta-analysis” published on June 22, 2020, in PLOS Neglected Tropical Diseases [1]. *Echinococcus granulosus sensu lato*, causing cystic echinococcosis (CE) and *Echinococcus multilocularis*, causing alveolar echinococcosis (AE) are transmitted to humans through the fecal-oral route. While identifying and quantifying the role of different routes of transmission to humans would improve control strategies, these information are very difficult to obtain for CE and AE [2].

The results from this systematic review and meta-analysis and from other 2 similar works on the same topic by Possenti and colleagues and Conraths and colleagues, also published in PLOS Neglected Tropical Diseases in 2016 and 2017, respectively [3,4], reach somehow different results. In Possenti and colleagues and Conraths and colleagues [3,4], dog-related factors (e.g., dog ownership, contact with dogs/playing with dogs) were not consistently associated with risk of CE. In the same studies, water-related and food-related factors were not associated with risk of CE, while having a kitchen garden and type of drinking water were associated with the risk of AE [3,4]. Differently, Torgerson and colleagues summarized their results by attributing consistently the majority of infections to “contact with dogs” and “contact with water” for both infections [1]. Interestingly, “living in endemic areas,” which could be interpreted as a proxy for the “environmental contact” of Torgerson and colleagues, was the only factor consistently associated with risk of CE in Possenti and colleagues (association not statistically supported with AE in Conraths and colleagues) but was eventually not analyzed directly by Torgerson and colleagues [1,3,4].

These observations stimulate a due reflection regarding the limits, and, therefore, the usefulness of the systematic review and meta-analysis approach applied to variables that are explored through questionnaires, for long-latent infectious diseases, such as CE and AE, which are usually discovered, if ever, years after the actual infection event has occurred, and that do not cause any acute symptom at the time of infection able to guide the source attribution.

While the use of questionnaires is the only way to investigate risk factors for diseases with the abovementioned characteristics, their substantial limits have been largely discussed in 2 recent papers [2,5] and imply that their results should be taken with caution, at least when used to issue practical recommendations on infection control measures. These include, among others, (1) recall bias; (2) bias deriving from the population sampling strategy; (3) difficulties in interpreting answers in terms of “common habit” versus “occasional behavior”; (4) failure to take into account the change of conditions or habits (e.g., dog ownership) over a long period of time; (5) type of management of conflicting/inconsistent answers among the questions of the questionnaire (which is virtually never made explicit in published papers); and (6) investigation of confounding or “proxy” factors rather than real pathways of transmission (e.g., using “feeding offal to dogs” as a proxy for “contact with dog,” which in turn does not explicitly or constantly mean “regularly touching the dog and then do not wash hands before direct or indirect contact with mouth,” which would be the actual pathway of eggs transmission to humans). Related to this latter issue is also the apparent tendency, in the field of echinococcal infections, to focus on a set of “usual” questions not necessarily related directly to transmission to humans but rather to the perpetuation of the parasite transmission cycle (e.g., slaughterhouse practices or dog ownership) as opposed to questions investigating habits corresponding to pathways by which a helminth egg can end up in a human mouth [2].

To add complexity and difficulties in deriving solid and meaningful data from such an approach, and, even more important, in trying to issue practical recommendations from the results of such studies, is the evidence that relevant differences may arise between results of equally well-conducted and rigorous systematic reviews and meta-analyses. This is the case for the 3 systematic reviews above mentioned [1,3,4] taken here as hints for discussion. Differences between the conclusions of the studies, which were all conducted with a rigorous methodology and basically superimposable inclusion criteria, may therefore derive from other factors. Firstly, the databases searched; for example, only 2 of the databases used in Possenti and colleagues [4] and Conraths and colleagues [3] were common to the databases searched by Torgerson and colleagues [1], and language limitations were different. Furthermore, the systematic review by Torgerson and colleagues [1] was carried out later than that of Possenti and colleagues [4] and Conraths and colleagues [3], implying the likely availability of more studies for the former review. Secondly, the restrictiveness of publication quality criteria applied for inclusion, which becomes particularly important when dealing with gray literature. Thirdly, the criteria for data extraction, pooling, and analysis applied for data meta-analysis, which may be more or less “reelaborated” and therefore may give a different “angle” to the final results of the analysis. For example, while Possenti and colleagues [4] and Conraths and colleagues [3] evaluated a large number of direct or indirect factors, Torgerson and colleagues [1] opted to fit different questions in each of their 4 investigated categories (direct contact with dog, water, food, and environment), by interpreting the meaning of different questions “on a case-by-case basis,” (e.g., “dog ownership,” “contact with dogs,” “feeding offal to dogs,” and “treatment of dogs” for the single risk factor “contact with infected dogs”). This last point of discussion has particular implications for the transfer of scientific results to practical recommendations. In the case of Torgerson and colleagues [1], for example, this grouping and interpretation of extracted data may be the reason why their estimates for what concerns foodborne attributable fractions (AF) for CE (AF 0.23, confidence interval (CI) 0.02 to 0.47), for instance, were actually the same of the figures provided by the expert elicitation of the FERG (Foodborne Disease Burden Epidemiology Reference Group of the WHO) study (around AF 0.2, interquartile range (IQR) <0.05% to 50%) [6], which, in the actual words of the authors, had CI “so wide to be largely uninformative” [1]. Interestingly, while the authors strongly conclude from their results that “dog contact [AF 0.26, CI 0.14 to 0.40] and drinking contaminated water [AF 0.29, CI 0.12 to 0.52] are major pathways of transmission of CE,” these figures seem, after all, not substantially different from the AF figures estimated for food [AF 0.23, CI 0.02 to 0.47] and environment [AF 0.21, CI −0.06 to 0.49]. Similar observations apply for AE. This lack of predominance of a pathway of transmission over the others may reflect that all these biologically plausible pathways [2] apply in equal manner, which would be perfectly possible, but also may imply that these estimation methodologies are based on insufficient and poorly informative data, which cannot result in any better estimate than a fair share between biologically plausible routes. On the other hand, the approach taken by Possenti and colleagues [4] and Conraths and colleagues [3], who evaluated a large number of direct or indirect factors, may have resulted in lower chances to individuate important pathways of infection and potential risk factors with statistical significance.

Finally, the importance of relating estimate figures with experimental data deserves a last reflection. As recently summarized for CE [2], very little and very heterogeneous data are available regarding the actual contamination by *Echinococcus* spp. eggs on dog’s fur, water, vegetables for human consumption, soil, and surfaces or fomites more in general. As stressed in this latter paper, while consumption of contaminated food and water, as well as contact or playing with dogs, are classically mentioned as the most important routes of human infection, experimental data just support their biological plausibility and possibility, without individuating an obvious predominance of one over the others. Actually, taken together, data suggest that local sociocultural and individual factors may have a dramatic influence on a route of transmission being predominant over another, making “global estimates” of odds ratio and AF de facto of very limited practical value beside public health advocacy.

To conclude, when investigating risk factors and related parameters for diseases such as CE and AE, results of analyses from questionnaire-based studies should be interpreted with caution. While these may be useful in ranking estimates for public health and advocacy purposes, their actual practical relevance for control programs or targeted hygiene education campaigns may be less consistent, if not openly misleading, when “general” concepts are applied uncritically in different epidemiological and sociocultural contexts.
